# A Rare Case of Aggressive Tongue Malignant Melanoma: Clinical Presentation and Treatment Approach

**DOI:** 10.7759/cureus.96184

**Published:** 2025-11-05

**Authors:** Merve Ozgen, Elmas Nur Ece Uygur, Candan Demiroz Abakay, Sibel Cetintas, Meral Kurt

**Affiliations:** 1 Radiation Oncology, Uludag University, Bursa, TUR

**Keywords:** adjuvant radiotherapy, case report, head and neck cancer, local control, oral mucosal melanoma, tongue melanoma

## Abstract

Tongue malignant melanoma is a very rare and highly aggressive subtype of oral mucosal melanoma (OMM), often diagnosed at advanced stages due to its asymptomatic course and rapid progression. This case report describes a 69-year-old female who presented with a progressively enlarging lesion on the ventral surface of her tongue, present for 10 years. Diagnosis of spindle cell malignant melanoma was confirmed by incisional biopsy, and staging revealed right cervical lymph node involvement without distant metastasis. The patient underwent hemiglossectomy, comprehensive neck dissection, and reconstructive surgery, followed by adjuvant radiotherapy (RT) with a simultaneous integrated boost (SIB) delivering 60 Gy to the surgical bed and high-risk lymph nodes and 54 Gy to contralateral low-risk lymphatics. Despite achieving initial local control (LC), aggressive distant metastases, including mediastinal lymph nodes, bilateral lung nodules, and bone lesions, rapidly developed within three months post-RT. Genetic analysis revealed no BRAF mutation, thus limiting targeted therapeutic options. This case underscores the significant diagnostic and therapeutic challenges posed by tongue malignant melanoma, emphasizing its aggressive biological behavior and high metastatic potential. A multidisciplinary approach combining radical surgery and adjuvant RT is crucial; however, the rapid distant progression highlights the critical need for more effective systemic treatment options and the integration of genetic profiling to guide personalized therapies. Further comprehensive research is essential to optimize management strategies and improve the limited survival outcomes associated with this rare and challenging malignancy.

## Introduction

Malignant melanoma is a neoplasm originating from melanocytes, which are derived from neural crest cells [1]. While these cells are typically located at the basal membrane of the epidermis, they can also be found in mucosal membranes, including those of the oral cavity [1]. Primary oral mucosal melanoma (OMM) arises from melanocytes within the oral mucosal basal membrane and accounts for approximately 0.8-3.7% of all melanomas and 0.5% of oral neoplasms [1-3]. Although the oral cavity is an uncommon site for melanoma overall, tongue involvement is exceptionally rare, accounting for only a small fraction of oral melanomas (<4%), with very few cases reported in the literature [3,4]. The most common anatomical sites include the hard palate (47%), gingival mucosa (27.6%), retromolar trigone, and buccal mucosa [4,5]. OMM usually presents at an older age compared to cutaneous melanoma, with most cases diagnosed between the ages of 50-80 years and a median age of 70 [5].

The etiology of OMM remains unclear. However, potential contributing factors include chronic mechanical trauma from dental prostheses, alcohol and tobacco use, and exposure to formaldehyde [6]. Treatment strategies often involve surgical resection, adjuvant radiotherapy (RT), concurrent chemoradiotherapy, and systemic chemotherapy [7]. Despite these interventions, the prognosis remains poor, with reported five-year overall survival (OS) rates ranging from 15% to 45% [8].

## Case presentation

**Figure 1:**
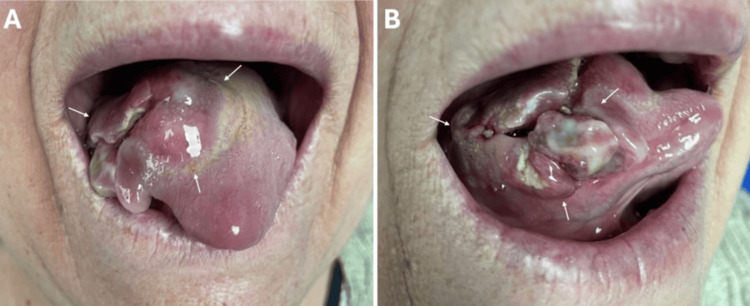
Preoperative tongue lesion Preoperative lesion is shown on the right ventral (A) and lateral (B) tongue surfaces. White arrows indicate the irregular and ulcerated mucosal mass on the tongue.

A 69-year-old female with a 10-year history of a tongue lesion that had recently increased in size presented to our institution. Her past medical history included hypertension and coronary artery disease. Clinical examination revealed an ulcerovegetative mass approximately 4 cm in diameter, extending from the right ventral tongue to the midline (Figures 1A, 1B). No palpable cervical lymphadenopathy or systemic findings were detected. Incisional biopsy confirmed a spindle cell subtype of malignant melanoma.

**Figure 2:**
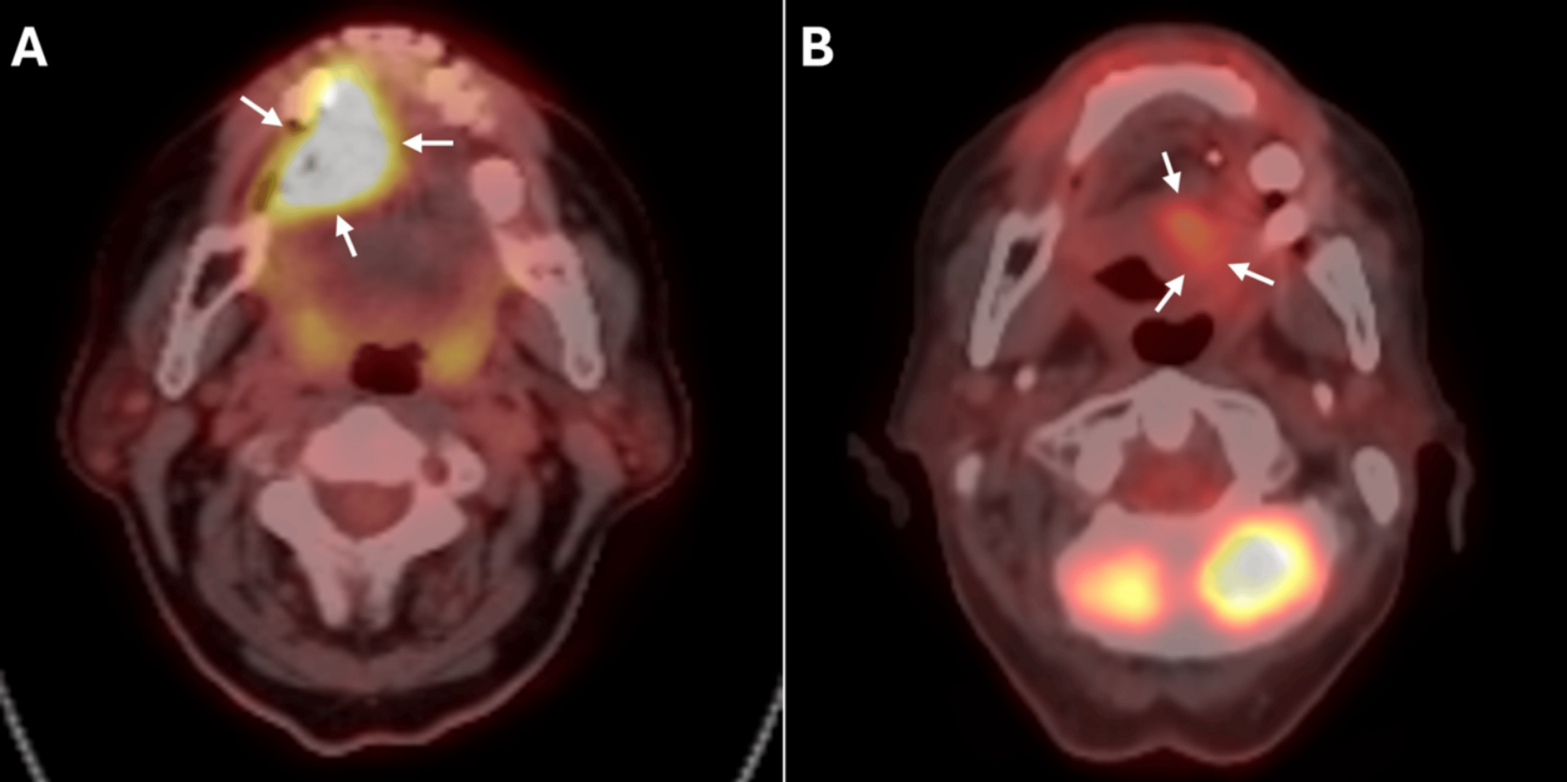
Preoperative and post-radiotherapy PET/CT findings Preoperative PET/CT showing a hypermetabolic primary tongue mass (A) and postoperative PET/CT at three months demonstrating mild metabolic activity at the surgical site consistent with post-treatment changes (B). White arrows indicate areas of FDG uptake.

**Figure 3:**
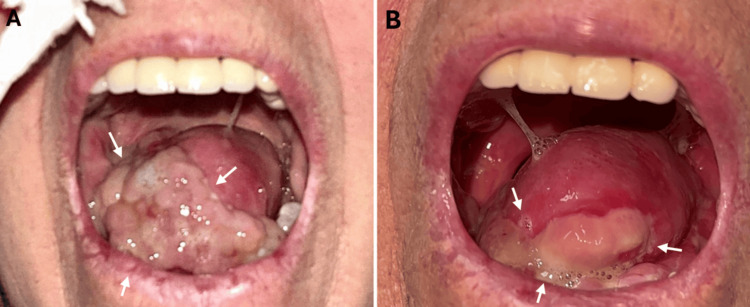
Flap site before and after radiotherapy The flap site is shown after hemiglossectomy before radiotherapy (A) and at the completion of radiotherapy (B). White arrows highlight the surgical flap margin and post-radiotherapy mucosal changes.

Staging with ¹⁸F-fluorodeoxyglucose (FDG) PET/CT demonstrated a hypermetabolic mass in the right anterior tongue (35 × 23 mm, SUVmax 9.32, Figure 2A), a hypermetabolic lymph node in right cervical level III (11 mm, SUVmax 3.68), and multiple small cervical lymph nodes with mild uptake; no distant metastasis was observed. The patient subsequently underwent right hemiglossectomy, right neck dissection (levels I-V), left neck dissection (levels I-III), tracheotomy, and reconstructive surgery (Figure 3A).

Histopathological evaluation revealed a 4.8 × 3.7 × 3.5 cm spindle cell melanoma with a tumor thickness of 1.7 cm. Lymphovascular invasion was absent, while perineural invasion and pigmentation were present. All surgical margins were negative. One metastatic lymph node (1.7 cm in diameter) was identified in the right level III without extranodal extension. Adjuvant RT was recommended by our institutional Head and Neck Cancer Tumor Board and subsequently administered. Concurrent chemotherapy was not recommended due to limited supporting evidence in mucosal melanoma. Genetic analysis was performed to evaluate potential targeted therapies.

**Figure 4:**
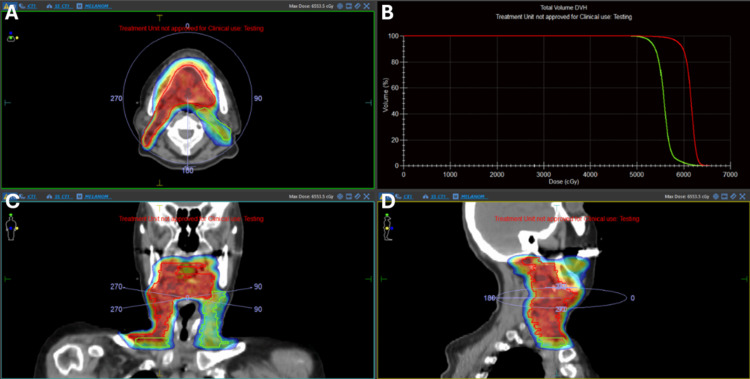
Radiotherapy planning images Radiotherapy planning images demonstrate high-risk (CTV60) and low-risk (CTV54) clinical target volumes, shown with dose color wash and the corresponding dose-volume histogram (DVH). A: axial view showing the high-dose region encompassing the primary tumor bed; B: dose-volume histogram (DVH) comparing coverage for CTV60 (red) and CTV54 (green); C: coronal view demonstrating superior-inferior extent of the target volumes; D: sagittal view illustrating anterior-posterior coverage of the planning target volumes.

For RT planning, CT simulation was performed using a thermoplastic mask and tongue depressor, with 2.5-mm slice thickness and no contrast. Target delineation was guided by preoperative PET/CT imaging and surgical notes. The high-risk clinical target volume (CTV) included the surgical bed, residual tongue, tongue base, floor of mouth musculature, and ipsilateral cervical levels I-IV. Contralateral cervical levels I-IV were defined as low-risk CTV. A simultaneous integrated boost (SIB) technique delivered 60 Gy in 2.0 Gy fractions to the high-risk CTV and 54 Gy in 1.8 Gy fractions to the low-risk CTV. Treatment planning was performed using Monaco v6.2.2.0 (Elekta AB, Stockholm, Sweden) with a volumetric-modulated arc therapy (VMAT) technique, employing two full arcs at couch angles of 9° and 351°, delivered with 6-MV photons on an Elekta Synergy linear accelerator (Elekta AB, Stockholm, Sweden) (Figure 4).

During RT, weekly clinical assessments, complete blood counts, and biochemical tests (including C-reactive protein (CRP)) were performed. On day 25 of treatment, the patient developed grade 2 dermatitis and grade 3 mucositis, according to the Common Terminology Criteria for Adverse Events (CTCAE) v5.0 criteria. RT was completed over 53 days. At the completion of RT, the flap site demonstrated post-treatment changes, including mucositis, erythema, and edema (Figure 3B). Post-treatment evaluation with contrast-enhanced neck CT at one month showed no evidence of residual or recurrent disease, with only mild postoperative changes. At 1.5 months post-RT, clinical examination revealed bilateral neck edema without palpable lymphadenopathy, and intraoral examination demonstrated no evidence of disease in the oropharynx or flap site. At three months post-treatment, ¹⁸F-FDG PET/CT showed mild metabolic activity at the surgical site consistent with post-treatment changes, suggesting good locoregional control (Figure 2B), whereas systemic evaluation revealed multiple hypermetabolic mediastinal lymph nodes, bilateral pulmonary nodules, and widespread osseous metastases. The patient was referred to medical oncology for systemic therapy evaluation.

## Discussion

Tongue malignant melanoma is a rare and aggressive subset of OMM, with limited literature and no standardized management guidelines [4,5,7]. Most OMM cases are thought to arise de novo; however, up to one-third of patients report longstanding pigmented oral lesions preceding diagnosis [9]. Our patient had a pigmented lesion that remained asymptomatic for a decade before exhibiting rapid enlargement in the months preceding diagnosis, highlighting the insidious course and diagnostic challenges of mucosal melanoma [4]. Consistent with prior reports, OMM typically presents at advanced stages with regional or distant metastases [4,5].

Surgical excision remains the cornerstone of therapy, often combined with neck dissection, RT, chemotherapy, or immunotherapy depending on stage and location [4,5,7]. Adjuvant RT is generally recommended for patients with positive surgical margins or other adverse risk features [10-13]. Multiple studies have demonstrated improved local control (LC) with RT, although an OS advantage has not been established [11-14]. In our case, perineural invasion and nodal metastasis were adverse prognostic factors, supporting the decision for adjuvant RT. Although systemic chemotherapy has been employed in advanced melanoma, response rates in mucosal melanoma are generally poor [4]. Therefore, in our patient, chemotherapy was not administered due to the limited expected benefit and the potential risk of toxicity.

Although initial locoregional control was achieved, distant metastases developed rapidly, reflecting the high systemic metastatic potential of mucosal melanoma. Krengli et al. reported that doses above 54 Gy improved LC but not OS [15]. Similarly, Owens et al. noted improved LC following adjuvant RT, though OS remained unaffected [13]. Our patient received 60 Gy to the surgical bed and high-risk lymph nodes and 54 Gy to contralateral lymph nodes, achieving early LC but failing to prevent systemic dissemination.

The genetic profile of mucosal melanoma differs substantially from cutaneous melanoma. Lower frequencies of KIT (14.6%), NRAS (5.6%), and BRAF (7%) mutations have been reported [4]. BRAF mutations, which predict responsiveness to targeted therapy, were absent in our patient. This highlights the biological distinction of mucosal melanoma and the limited applicability of established targeted regimens in this population.

Prognosis remains poor despite multimodal management. Meleti et al. reported five-year OS rates of 17.2% in head and neck mucosal melanoma and 38.4-41.6% in OMM, regardless of RT [16]. Aggressive follow-up and early systemic therapy evaluation are therefore critical. According to Memorial Sloan Kettering Cancer Center, a tumor thickness of more than 5 mm, the presence of lymphovascular invasion, advanced stage at presentation, and the development of distant metastases are independent adverse prognostic factors in mucosal melanoma [17].

Given its aggressive course, tongue malignant melanoma requires a multidisciplinary approach that integrates surgery, adjuvant therapy, and molecular profiling. This case underscores the diagnostic challenges, the potential role of adjuvant RT, the importance of genetic evaluation, and the necessity of integrating systemic therapy into management strategies to address the high risk of distant metastases. Further multicenter studies are urgently needed to refine management strategies and improve outcomes in this rare and lethal disease.

## Conclusions

Tongue malignant melanoma is a rare and aggressive malignancy with a poor prognosis. This case highlights the diagnostic challenges, the need for surgical resection with adjuvant RT, and the limitations of current systemic options. Despite achieving LC, early distant metastases occurred. In addition to highlighting the aggressive nature of tongue malignant melanoma, this case underscores the limitations of current systemic therapeutic strategies, particularly in achieving long-term disease control. The rapid progression despite optimal local therapy draws attention to the urgent need for novel systemic agents and individualized treatment approaches guided by molecular profiling. This report may contribute to the limited literature on mucosal melanomas of the head and neck and prompt further investigation into optimal multimodal management and follow-up strategies.
